# The relevance of using in situ carbon and nitrogen data and satellite images to assess aboveground carbon and nitrogen stocks for supporting national REDD + programmes in Africa

**DOI:** 10.1186/s13021-019-0127-7

**Published:** 2019-09-10

**Authors:** Adéyèmi Chabi, Sven Lautenbach, Jérôme Ebagnerin Tondoh, Vincent Oladokoun Agnila Orekan, Stephen Adu-Bredu, Nicholas Kyei-Baffour, Vincent Joseph Mama, John Fonweban

**Affiliations:** 1 Department of Geography, University of Parakou, Parakou, Republic of Benin; 2 West African Science Service Centre On Climate Change and Adapted Land Use (WASCAL), Competence Centre Ouagadougou, 06 BP 9507 Ouaga 06 Ouagadougou, Burkina Faso; 3 GIScience Research Group, Institute of Geography, University of Heidelberg, Mathematikon, Berliner Str. 45, 4. OG, Raum 004, Heidelberg, Germany; 4 Institute of Geodesy and Geoinformation, University of Bonn, Nussallee 1, 53115 Bonn, Germany; 5 Department of Geography, University of Abomey-Calavi, BP 677 Abomey-Calavi, Republic of Benin; 6 CSIR-Forestry Research Institute of Ghana, Tryft, University P.O. Box 63, Kumasi, Ghana; 7 Departments of Agricultural Engineering, The College of Engineering, Kwame Nkrumah University of Science and Technology, Kumasi, Ghana; 8 National Institute for Agricultural Research of Benin (INRAB), 06 BP 1105 Cotonou, Benin; 9 Food & Agriculture Organization of the United Nations (FAO), Regional Office for Africa (RAF), Accra, Ghana

**Keywords:** Relevance, In situ, Carbon, Nitrogen, Assess, Aboveground, REDD + programmes, Africa, Sudan Savannah

## Abstract

**Background:**

To reduce the uncertainty in estimates of carbon emissions resulting from deforestation and forest degradation, better information on the carbon density per land use/land cover (LULC) class and in situ carbon and nitrogen data is needed. This allows a better representation of the spatial distribution of carbon and nitrogen stocks across LULC. The aim of this study was to emphasize the relevance of using in situ carbon and nitrogen content of the main tree species of the site when quantifying the aboveground carbon and nitrogen stocks in the context of carbon accounting. This paper contributes to that, by combining satellite images with in situ carbon and nitrogen content in dry matter of stem woods together with locally derived and published allometric models to estimate aboveground carbon and nitrogen stocks at the Dassari Basin in the Sudan Savannah zone in the Republic of Benin.

**Results:**

The estimated mean carbon content per tree species varied from 44.28 ± 0.21% to 49.43 ± 0.27%. The overall mean carbon content in dry matter for the 277 wood samples of the 18 main tree species of the region was 47.01 ± 0.28%—which is close to the Tier 1 coefficient of 47% default value suggested by the Intergovernmental Panel on Climate Change (IPCC). The overall mean fraction of nitrogen in dry matter was estimated as 0.229 ± 0.016%. The estimated mean carbon density varied from 1.52 ± 0.14 Mg C ha^−1^ (for Cropland and Fallow) to 97.83 ± 27.55 Mg C ha^−1^ (for *Eucalyptus grandis* Plantation). In the same order the estimated mean nitrogen density varied from 0.008 ± 0.007 Mg ha^−1^ of N (for Cropland and Fallow) to 0.321 ± 0.088 Mg ha^−1^ of N (for *Eucalyptus grandis* Plantation).

**Conclusion:**

The results show the relevance of using the in situ carbon and nitrogen content of the main tree species for estimating aboveground carbon and nitrogen stocks in the Sudan Savannah environment. The results provide crucial information for carbon accounting programmes related to the implementation of the REDD + initiatives in developing countries.

## Background

In the context of climate change issues, emissions from deforestation and forest degradation in developing countries constitute some 20 percent of the total global emission of greenhouse gases annually [1]. Thus, reducing emissions from deforestation and degradation, biodiversity conservation, sustainable forest management and enhancement of forest carbon stocks (REDD +) in developing countries has become important frameworks to mitigate climate change and limit the rise in global temperature to no more than 2 °C [1–3]. Current challenges for the management of forests and other land use classes are the development of verifiable, reliable, accurate and cost-effective methods to adequately document forest resources dynamics [2]. The estimation of aboveground carbon stocks and the related uncertainties arise from inadequate data [3, 4]. These uncertainties in turn compromise the estimation of terrestrial carbon emissions as well as the knowledge of in situ data [3, 5–7]. Better assessments of aboveground nitrogen stocks could also be of interest since they provide necessary information for predicting nitrous oxide emission from damaged or burned trees. The accuracy of the estimation of mean carbon and nitrogen density for each land use/land cover class depends thereby on reliable carbon and nitrogen content estimates per main tree species, species frequency estimates per land use/land cover class and the availability of reliable allometric models to infer oven-dry aboveground biomass of trees from tree census data [8].

Allometric equations have been used by many authors all over the world [8–19, 58, 59] for estimating biomass stocks of ecosystems. The estimation of carbon stocks in Sub-Saharan Africa is based on allometric models and forest inventory data [8, 20–31]. Many studies so far focused on the estimation of aboveground biomass of forest ecosystems, specific tree species or plantations [8, 20, 22, 23, 25, 27, 32–40, 60]. The study from Kuya [29] was few of them which focused on the estimation of aboveground biomass in agricultural landscapes. However, woody vegetation in agricultural landscapes represents a significant carbon pool. In sub-Saharan Africa, the majority (87%) of agriculturally dominated landscapes has a tree cover of more than 10% [41].

To reduce the uncertainty in estimates of carbon dioxide and nitrous oxide emissions from deforestation and forest degradation, more complete and higher quality information-based satellite images and in situ data is needed. The estimation of the total carbon and nitrogen stocks at the landscape level is complex since the vegetation pattern changes from one land use/land cover class to another and the tree species distribution varies gradually by size and species. Additionally, there is a need for reliable methods that are applicable to target species in the region of interest [41]. With increasing data requirements and analytical complexity from Tier 1 to Tier 3, the accuracy and precision of the carbon estimate also increases [42]. An accurate estimation of aboveground carbon and nitrogen stocks is recommended by the IPCC [42] to considerably reduce the uncertainty in the Tier 3 approach. The Tier 1 approach [42] suggested a coefficient of 0.47 to convert mean biomass density to the mean carbon density for a defined ecosystem or land use/land cover class. This default value is applied in many cases at the national level by many developing countries in the absence of information on carbon content of the main tree species of the region. In some cases a coefficient of 0.5 has been applied [4, 43]. Both default values may underestimate or overestimate the carbon stock, leading to a substantial level of uncertainty. In addition to information on regional land use, specific conversion factors and allometric models are needed that allow a biomass estimation at the landscape scale based on properties that are easy and reliable to measure under field conditions. Conversion factors and allometric models can then be used together with remote sensing based land use/land cover information to estimate the current carbon and nitrogen stocks or to quantify the changes in these stocks.

The aim of this study was to quantify the aboveground carbon and nitrogen stocks at the landscape level for the current (2013–2014) land use/land cover at the scale of a watershed in the West African Sudan Savannah using in situ carbon and nitrogen content of the main tree species of the site.

## Results and discussion

### Carbon and nitrogen content of dry matter of the main tree species

**Fig. 1:**
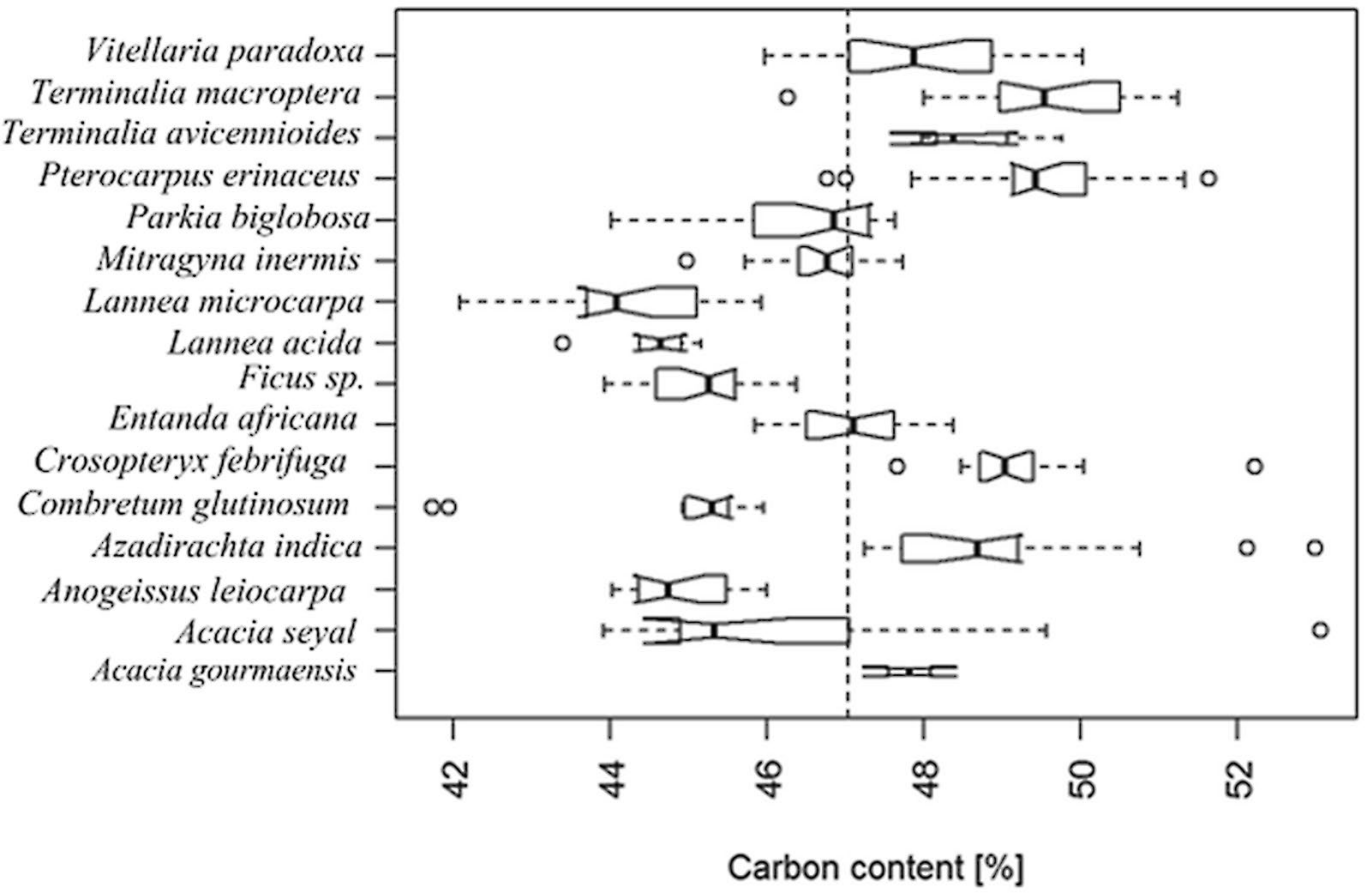
Boxplot showing the distribution of carbon content in dry matter per tree species. The dashed vertical line shows the overall mean which is closed to the IPCC Tier 1 default value of 47%

The fraction of carbon and nitrogen in the dry matter of the wood samples of the main tree species of the Dassari watershed in this Sudan Savannah environment differed clearly between the different tree species (Table 1, Fig. 1). The tree species with a high mean carbon fraction were *Terminalia macroptera* (49.43 ± 0.24%), *Pterocarpus erinaceus* (49.43 ± 0.27%) and *Crosopteryx febrifuga* (49.17 ± 0.21%). The lowest carbon content of dry matter was obtained for C*ombretum glutinosum* (min 41.73%) with the mean of the species of 44.72 ± 0.44% and the highest for *Acacia seyal* (max 53.07%) with the mean of the species of 46.50 ± 0.68%. The estimated mean per tree species varied from 44.28 ± 0.21% to 49.43 ± 0.27%. The overall mean of the 277 stem wood samples for all species was 47.01 ± 0.28% which is in line with default value of 47% in the IPCC [42] Tier 1 approach. The substantial variation of carbon content across tree species confirmed the relevance of using in situ carbon content of the main tree species of the region (higher tier) for carbon accounting.

**Table 1:** Carbon (C) and nitrogen (N) contents of stem wood of the main tree species of the watershed

Carbon (C) contents (% dm)					Nitrogen (N) contents (% dm)			DBH (cm)		C/N ratio		
Trees species	n	Min	Max	Mean (SE)	Min	Max	Mean (SE)	Min	Max	Min	Max	Mean (SE)
*Terminalia macroptera*	19	46.267	51.241	49.474 (0.266)	0.108	0.303	0.192 (0.013)	9.3	40.7	160.50	428.39	281.81 (18.33)
*Terminalia avicennioides*	03	47.971	49.759	48.70 (0.53)	0.155	0.181	0.168 (0.007)	16.6	24	265.03	312.16	289.96 (13.67)
*Acacia seyal*	14	43.928	53.071	46. 50 (0.684)	0.13	0.583	0.290 (0.037)	7.6	34.4	80.71	357.6	194.24 (21.72)
*Acacia gourmaensis*	02	47.55	48.09	47.824 (0.269)	0.297	0.349	0.323 (0.025)	13.4	19	160.11	137.80	148.95 (11.15)
*Combretum glutinosum*	11	41.737	45.959	44.72 (0.438)	0.14	0.358	0.241 (0.020)	8	32	125.94	320.95	201.36 (19.15)
*Pterocarpus erinaceus*	21	46.779	51.645	49.438 (0.278)	0.164	0.427	0.242 (0.014)	6.9	44.7	110.09	295.09	216.28 (10.63)
*Anogeisus leiocarpus*	16	44.037	46.003	44.917 (0.167)	0.08	0.273	0.128 (0.012)	6.9	32.4	161.30	570.05	386.52 (28.28)
*Mitragyna inermis*	18	44.978	47.74	46.724 (0.174)	0.177	0.354	0.243 (0.011)	7	34.5	129.46	262.19	199.40 (9.23)
*Lannea microcrapa*	20	42.091	45.938	44.282 (0.209)	0.148	0.405	0.273 (0.015)	7	50.3	110.95	306.08	173.47 (11.14)
*Lannea acida*	6	43.408	45.164	44.526 (0.248)	0.14	0.386	0.265 (0.035)	10.8	36	115.60	320.80	186.92 (30.61)
*Ficus sp*	21	43.931	46.38	45.153 (0.139)	0.16	0.427	0.294 (0.015)	8.6	52.7	105.3	286.90	163.14 (9.83)
*Crosopteryx febrifuga*	18	47.662	52.229	49.172 (0.217)	0.118	0.306	0.182 (0.014)	5.6	30.6	161.14	417.54	295.68 (20.50)
*Entada Africana*	15	45.852	48.377	47.098 (0.191)	0.242	0.475	0.357 (0.016)	8.4	27.6	100.09	196.18	135.97 (6.75)
*Parkia biglobosa*	23	44.02	47.636	46.516 (0.214)	0.127	0.396	0.201 (0.013)	8.6	62.4	119.40	358.43	247.85 (12.35)
*Vitelaria paradoxa*	22	45.972	50.032	47.942 (0.228)	0.13	0.337	0.228 (0.010)	8	60	136.41	367.23	220.11 (11.37)
*Azadirachta indica*	16	47.253	52.999	49.005 (0.413)	0.104	0.302	0.177 (0.014)	8.8	50.5	162.43	474.64	302.38 (22.53)
*Anacardium occidentale*	25	44.928	47.693	46.446 (0.138)	0.103	0.32	0.161 (0.011)	9.2	57.9	146	441.34	375.79 (17.58)
*Eucalyptus grandis*	7	47.018	49.031	47.744 (0.350)	0.125	0.191	0.157 (0.011)	5.7	29.2	247.25	376.14	310.57 (21.94)

% dm, percentage of C and N in dry matter; n, number of selected trees. The stem wood samples of selected trees were extracted at 1.3 m of the ground. DBH range, range of diameter at breast height of sampled tree species. Figures in bracket represent the standard error (SE) of the mean

When applying the coefficient 0.5 as used by Chave et al. [8], Baccini et al. [4] to convert the mean biomass density into the mean carbon density for each LULC, the mean carbon density was overestimated for all LULC classes by 5.52% for Riparian forest and woodland, by 6.54% for Savannah Woodland, by 6.41% for Shrub Savannah, by 8.21% for grassland, by 7.6% for Cropland and Fallow, by 5.53% for Settlements, by 7.65% in Agroforestry systems and by 4.72% in Plantations. The application of the IPCC [42] default Tier 1 coefficient of 0.47 slightly overestimated carbon density by 0.15% (for Savannah Woodland), 0.54% (for Shrub Savannah), 1.72% (for grassland), 1.14% (for Cropland and Fallow), and 1.19% (for Agroforestry system) and underestimated by 0.81% (for Riparian forest and woodland), 0.80% (for Settlements) and 1.55% (for Plantation). We therefore recommend the use of the coefficient of 0.47 if one has to stick to the Tier 1 approach for carbon accounting in the Sudan Savannah environment.

The obtained carbon content for the most abundant species was in the same order of magnitude as results published by Guendehou et al. [37]; Andreae et al. [54]; Lasco et al. [55], Feldpausch et al. [56] and McGroddy et al. [57] even if the most abundant tree species varied considerably across the regions of the different case studies.

The nitrogen fraction of dry mater of the main tree species varied from 0.08% to 0.58%. The lowest mean nitrogen content for a single tree species was 0.128 ± 0.012% and the highest mean for a single tree species was 0.357 ± 0.016%. The overall mean fraction of dry matter of nitrogen content was 0.229 ± 0.016%. The species with highest nitrogen content in dry matter were *Acacia seyal, Acacia gourmensis, Ficus sp, Entanda Africana* and *Lannea microcarpa*. Human disturbance that affects these species could therefore lead to potentially high levels of N_2_O emissions with high global warming potential due to the high fraction of nitrogen content into the dry matter of their stem wood. The C/N ratio per tree ranged from 80.71 to 570.05. The mean C/N per tree species ratio ranged from 135.97 ± 6.75 to 386.52 ± 28.28 for the different species for all land uses.

### Carbon and nitrogen density and stocks at the landscape level

For the year 2013 the estimated stock in the watershed were for carbon 175,347.75 ± 10,735.95 Mg and for nitrogen 875.53 ± 51.76 Mg. The carbon density in Mg C ha^−1^ were 44.81 ± 2.38 (for Riparian forest and woodland), 21.35 ± 1.16 (for Savannah Woodland), 6.57 ± 0.35 (for Shrub Savannah), 1.67 ± 0.15 (for Savannah grassland), 1.52 ± 0.14 (for Cropland and Fallow), 2.30 ± 0.48 (for Settlements), 21.39 ± 6.68 (for Agroforestry system) and 97.83 ± 27.55 (for Plantation) (Table 2). The carbon density was higher in settlements than in croplands and Savannah grasslands which is in line with our field observation that Biali community in this region tends to plant mostly trees species like *Azadirachta indica* within the settlements that are characterized by a high carbon density. Carbon density was higher in riparian forest and woodland than in cashew plantations. Both carbon content (46.45 ± 0.14%) and tree density (300 trees per ha) was much lower in cashew plantations (*Anacardium occidentale*) compared to riparian forests and woodlands (1397 trees per ha). This implies that the carbon offset when clearing a patch of riparian forest and woodland for farming activities unfortunately cannot be compensated by cashew plantations. We estimated this loss as 23.42 Mg C ha^−1^. Despite the loss, it is important to adopt agroforestry after clearance of riparian forest since carbon loss is nearly twice as high for the conversion to cropland (44.81 ± 2.38 Mg C ha^−1^). If Savannah woodland is converted to cashew plantations differences in mean carbon density are low while the conversion to cropland leads for both Savannah Woodland and for shrub Savannah to a net loss in carbon. Plantations with *Eucalyptus grandis, Tectona grandis, Azadirachta indica* had higher carbon densities per ha than riparian forests and could therefore be used to compensate carbon emissions from land clearing. The use of *Gmelina arborea* in plantations compensates due to the low carbon density only partially for carbon emissions from land clearing.

**Table 2:** Mean carbon density (Mg C ha^−1^) and total carbon stocks (Mg C) by LULC class at the watershed scale

LULC/LUCa	Descriptive statistic			
	Range of carbon density (Mg C ha^−1^)		Mean carbon density (SE)	Total carbon stock (Mg C) (SE)
	Min	Max		
Forest land				159,841.01 ± 8721.48
Riparian forest and woodland	35.46	57.27	44.81 (2.38)	15,291.86 ± 813.16
Savannah Woodland	12.50	31.90	21.25 (1.16)	116,401.70 ± 6397.49
Shrub Savannah	2.76	12.22	6.57 (0.35)	28,147.43 ± 1510.82
Grassland				161.55 ± 15.23
Savannah grassland	0.03	2.98	1.67 (0.15)	161.55 ± 15.23
Cropland				12,272.24 ± 2326.92
Cropland and Fallow	0.03	4.33	1.52 (0.14)	12,272.24 ± 2326.92
Settlements				1125.66 ± 1187.20
Settlements	0.41	4.57	2.30 (0.48)	1125.66 ± 1187.20
Agroforestry				442.91 ± 138.47
Cashew plantation	4.99	98.08	21.39 (6.68)	442.91 ± 138.47
Plantation				1504.36 ± 435.29
*Eucalyptus grandis*	3.67	331.91	97.83 (27.55)	1346.27 ± 379.15
*Tectona grandis*	16.52	108.70	82.62 (33.09)	74.36 ± 29.78
*Azadirachta indica*	31.58	117.87	88.02 (28.23)	63.37 ± 0.32
*Gmelina arborea*	4.88	16.16	11.82 (3.50)	20.34 ± 6.02

The age of plantations and agroforestry system varied from 5 to 45 years old explained the large standard error (SE) and the large variance relative to the mean obtained from these plots. The area of each LULC class was provided in the Table 4

**Fig. 2:**
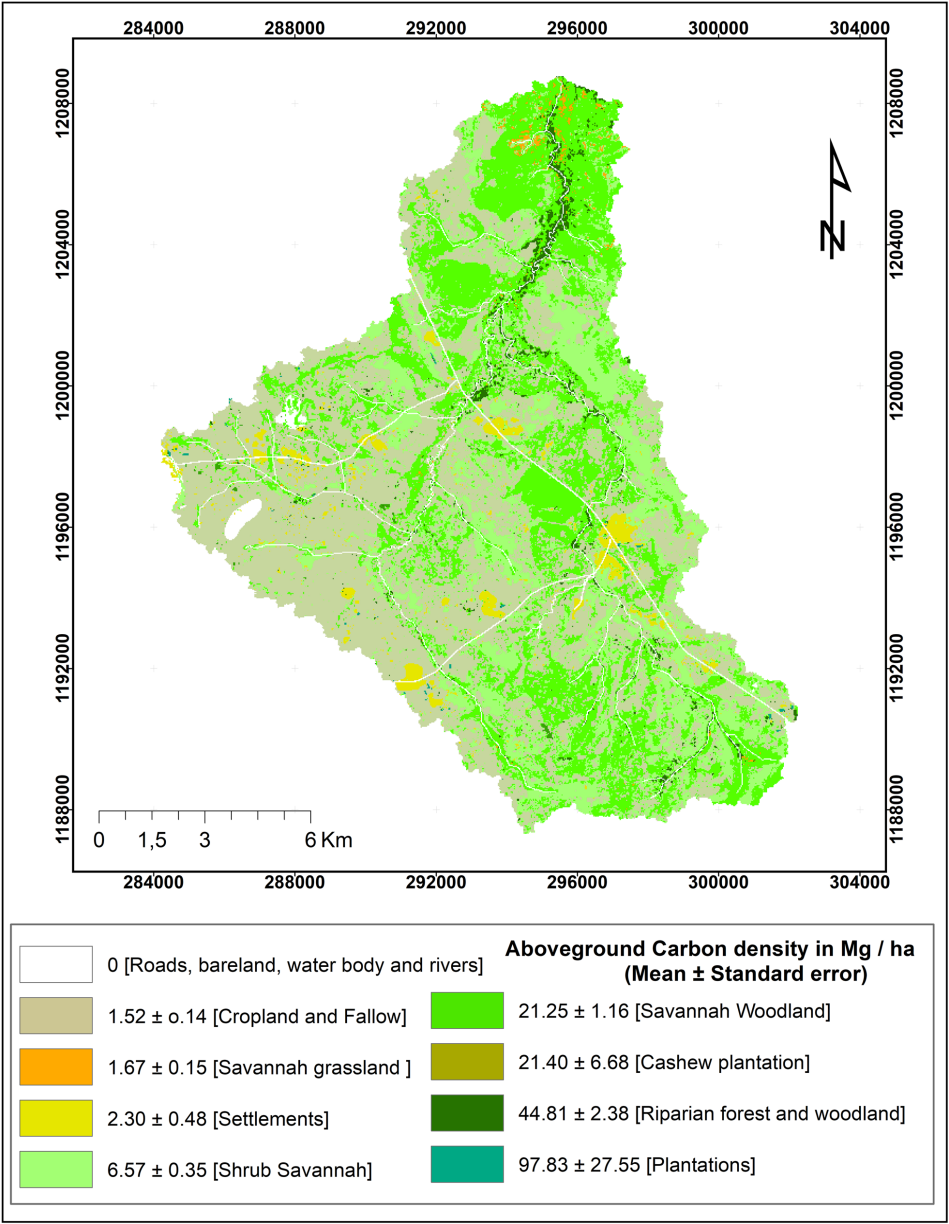
Carbon stocks at the watershed level in 2013. The classes correspond to the land use/land cover classes—i.e. each land use/land cover class is represented by a different class in the legend

**Fig. 3:**
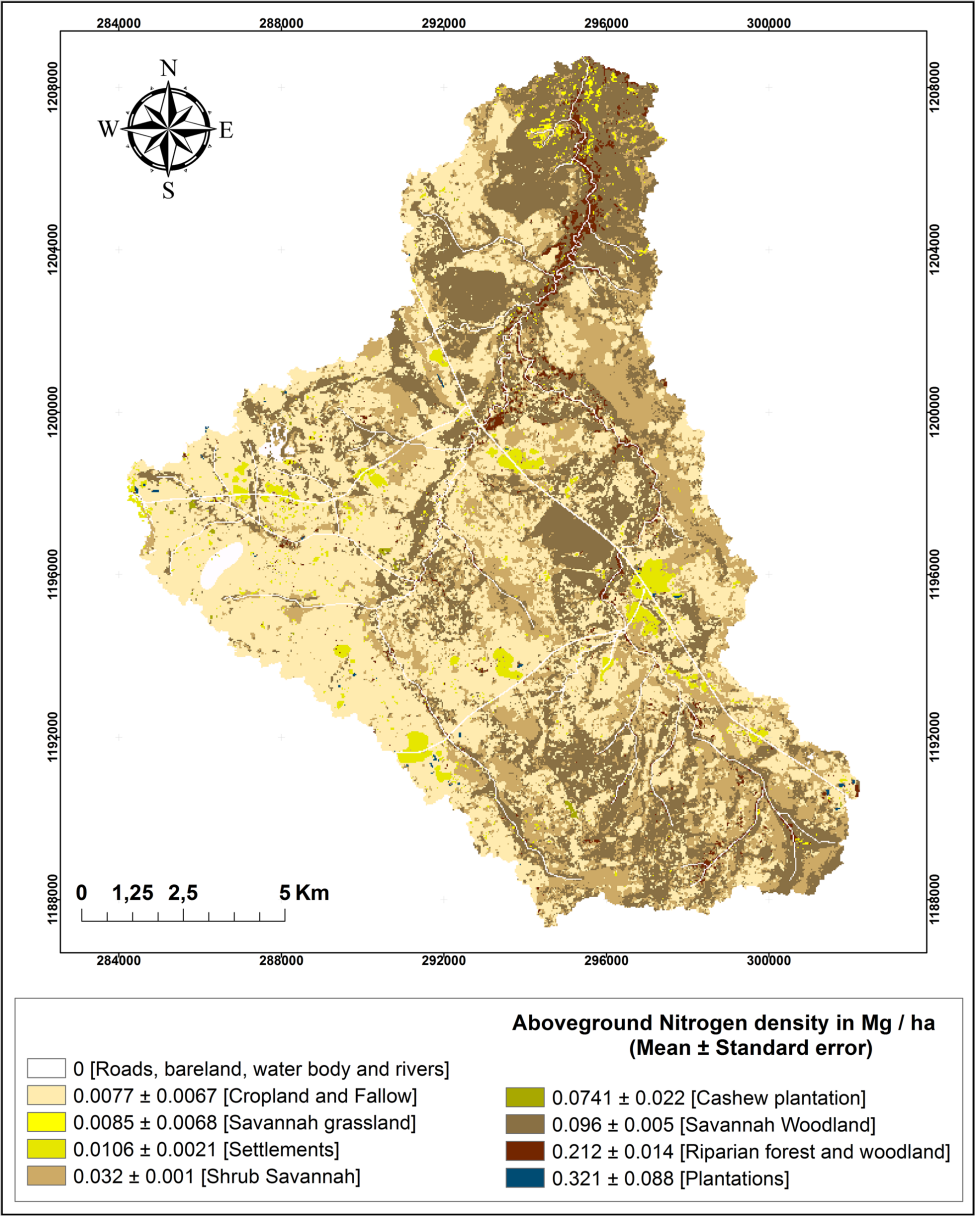
Nitrogen stocks at the watershed level in 2013. The classes correspond to the land use/land cover classes—i.e. each land use/land cover class is represented by a different class in the legend

For nitrogen (Table 3) relative effects of land use conversion were of similar magnitude as for carbon. Thus, the absolute differences are very different, but the relative differences are comparable. The different carbon and nitrogen density of the land use classes is reflected in the heterogeneous spatial distribution of carbon and nitrogen stocks at the watershed scale (Figs. 2, 3).

**Table 3:** Mean nitrogen density (Mg ha^−1^ of N) and total nitrogen stocks (Mg of N) by LULC class at the watershed scale

LULC/LUCa	Descriptive statistic			
	Range of nitrogen density (Mg ha^−1^ N)		Mean nitrogen density (Mg ha^−1^N) (SE)	Total nitrogen stocks (Mg N) (SE)
	Min	Max		
Forest land				740.37 ± 0.021
Riparian forest and woodland	0.170	0.285	0.212 (0.014)	72.41 ± 0.014
Savannah Woodland	0.045	0.160	0.096 (0.005)	530.79 ± 0.005
Shrub Savannah	0.008	0.064	0.032 (0.001)	137.16 ± 0.001
Grassland				0.825 ± 0.0006
Savannah grassland	0.0001	0.0178	0.0085 (0.0068)	0.825 ± 0.0.0006
Cropland				62.57 ± 0.0006
Cropland and Fallow	0.00018	0.0252	0.0077 (0.0067)	62.57 ± 0.0006
Settlements				5.20 ± 0.002
Settlements	0.0017	0.0201	0.0106 (0.0021)	5.20 ± 0.002
Agroforestry				1.53 ± 0.022
Cashew plantation	0.017	0.340	0.0741 (0.022)	1.53 ± 0.022
Plantation				5.01 ± 0323
*Eucalyptus grandis*	0.012	1.091	0.321 (0.088)	4.42 ± 0.088
*Tectona grandis*	0.058	0.418	0.291 (0.115)	0.26 ± 0.115
*Azadirachta indica*	0.114	0.425	0.317 (0.101)	0.23 ± 0.101
*Gmelina arborea*	0.024	0.079	0.058 (0.017)	0.10 ± 0.017

The age of plantations and agroforestry system varied from 5 to 45 years which explained the large standard error (SE) and the large variance relative to the mean obtained from their plots data. The area of each LULC class was provided in the Table 1

## Conclusion

The results showed the relevance of using the in situ carbon and nitrogen content of the main tree species in estimating aboveground carbon and nitrogen stocks in the Sudan Savannah environment. By assessing the carbon and nitrogen fraction in dry matter of main tree species of the region uncertainty could be substantially reduced by 0.15 to 1.72% lower and 0.80 to 1.55% higher compared to the default IPCC [42] Tier 1 value of 47% depending the land use/land cover class. The overall mean carbon content across all land use categories as the average of 277 wood samples for all species was 47.01% indicating that a Tier 1 value 47% instead of the sometimes used value of 50% should be used in the Sudan Savannah environment if no more detailed information is present. Both results on carbon and nitrogen density in each LULC class, and the carbon and nitrogen content per trees species provide important information for carbon accounting related to the implementation of national REDD + programmes of developing countries in the Sudan Savannah environment. Carbon stocks per ha in croplands and settlements in the case study region were comparable to Savannah grassland. Carbon stocks per ha in cashew plantations were comparable to Savannah Woodland but lower than riparian forests. The highest carbon stocks per ha were observed for plantations based on *Eucalyptus grandis, Tectona grandis, or Azadirachta indica*. While plantations of these three trees not endemic to West Africa are able to compensate carbon loss due to land use change trade-offs with other ecosystem goods and services and biodiversity should be considered.

Since the study took place at the local scale there is a need for the engagement of such work at the regional scale to confirm the importance of using in situ carbon and nitrogen data for carbon accounting. In this situation regional allometric equations are also of great importance for carbon accounting for the West African countries.

## Materials and methods

### Case study location

**Fig. 4:**
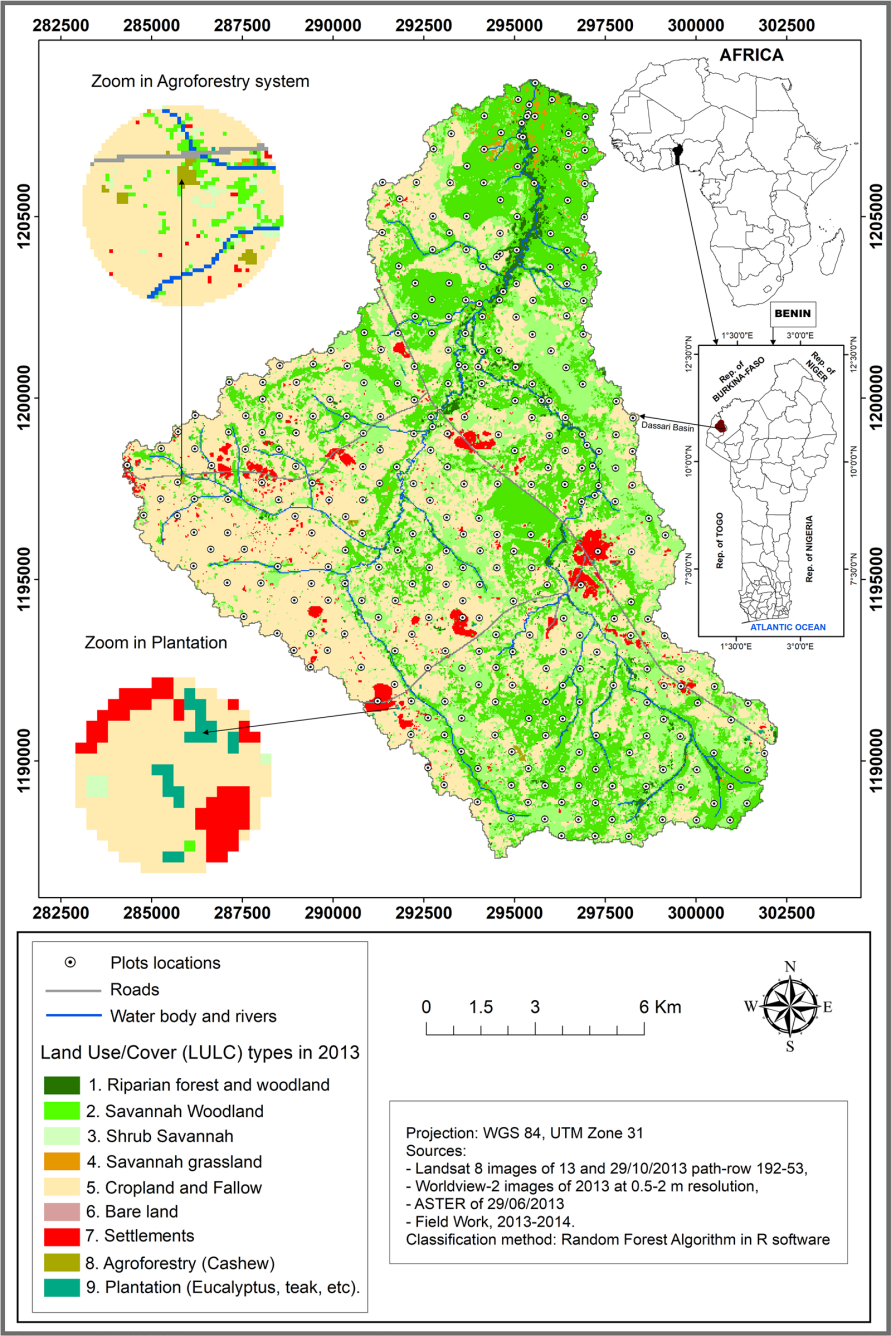
Study area and land use/cover map of 2013/2014 with plots locations

The region is located between 10°44′08″ N–10° 55′ 42″N and 1° 01′ 32″E–1°11′30″E, specifically at the Dassari Basin situated in the North-West of Benin (Fig. 4) whith a coverage area of 192.57 km^2^. Long-term (1952–2010) minimal daily temperature ranged from 15.25 to 25.08 °C with an average of 20.53 °C. Daily maximum temperature ranged from 26.63 to 39.27 °C with a mean temperature of 32.59 °C. Long-term (1971–2013) mean annual rainfall was 1054.94 mm. The region was characterized by two periods of extreme droughts (1978–1979; 1985–1986) and some moderate to severe drought using the standardized precipitation index (SPI) programme developed by Mckee [44].

### Methods

#### Image classification

We coupled two scenes of Landsat 8 (http://glovis.usgs.gov) together with ground truthing information to classify land use/land cover. Landsat 8 satellite images from 13 October 2013 and 29 October 2013 were used—both with path-row 193-53. October was chosen since photosynthetic activity of natural vegetation and crops is high and cloud cover and fire pattern disturbance tend to be minimized during that part of the year.

**Fig. 5:**
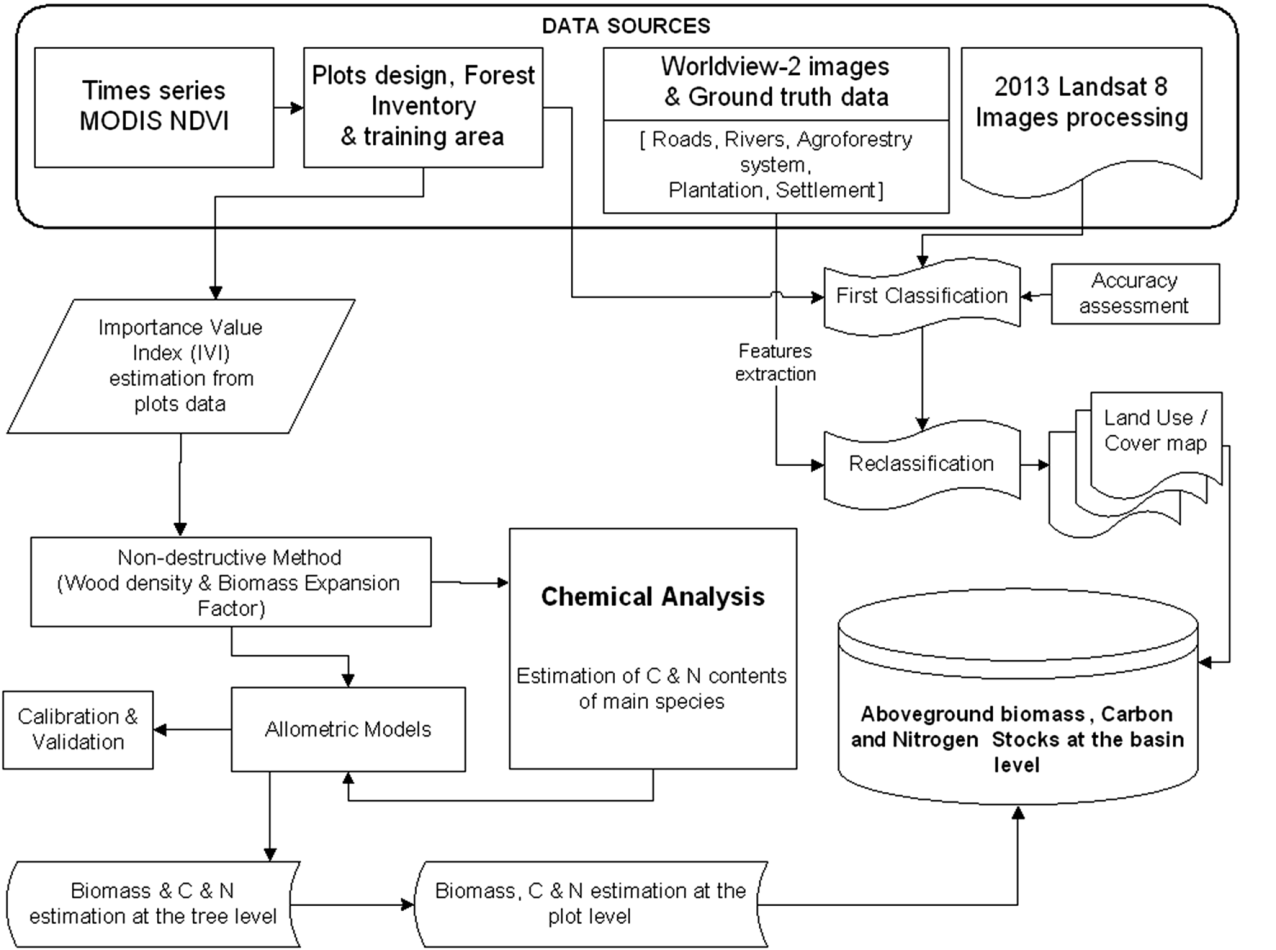
Flowchart of main steps for the assessment of the vegetation carbon and nitrogen stocks

Since it was not possible to separate agroforestry, forest land and plantations at the scale of the Landsat 8 data, these classes were separated based on several Worldview-2 (http://www.digitalglobe.com) imagery scenes with 0.5–2 m resolution together with additional ground truth data from known agroforestry and plantation plots to discriminate agroforestry system and plantation from natural vegetation (cf. Fig. 5).

Based on the ground truthing data derived for the sample points (cf. Fig. 4), a random forest [46, 47] model was trained and used to classify the Landsat 8 data. The analysis was done in R [48] using the package random Forest [49]. The accuracy of the classification (Fig. 5) was acceptable to good as indicated by the overall accuracy of 0.75 and the kappa index of 0.70 [50].

#### Forest inventory

In reference to the objective of the current study we focused our measurements in the stand tree species of each LULC (land use land cover) of the site (Table 4). During the forest inventory we found some tree species such as *Vitelaria paradoxa*, *Parkia biglobosa, Lannea microcrapa* and *Lannea acida* which have the economic value for farmers and which were not burnt or cut off. The same remark is applicable to savannah grassland where we also have stand tree in low density. According to Zomer et al. [41] in sub-Saharan Africa, the majority (87%) of agriculturally dominated landscapes has a tree cover of more than 10%. For this purpose the measurements (DBH and Height) of stand tree species which are within crop land and fallow and savannah grassland are also of concerned like others LULC (Table 1) in this study.

**Table 4:** Land use/land cover (LULC) classes and number of established plots

LUCa/LULC	Area (ha)	Percentage (%) in the basin	Area sampled (ha)	Number of established plots
Forestland				
Riparian forest and woodland	320.40	1.66	0.81	9
Savannah Woodland	5447.79	28.29	2.43	27
Shrub Savannah	4241.88	22.03	5.04	56
Grassland				
Savannah Grassland	96.48	0.50	3.06	34
Cropland				
Cropland and Fallow (Agricultural land)	8031.15	41.70	7.20	80
Settlement				
Settlement	486.72	2.53	8.00	8
Others land use				
Agroforestry	20.70	0.11	0.26	13
Plantation	16.74	0.09	0.46	23

NB: Agroforestry and plantation were identified as potential mitigation strategies to climate change. We therefore discriminated them from cropland. Percentage values do not add up to 100% since the basin also includes water bodies, roads and bare land that were not included in the table

Forest inventory was carried out from March to September 2014 in every LULC class. The plots were installed randomly proportionally to the area covered by the LULC class (Table 4) based on the equation of Pearson et al. [51]. The size of the plots was 30 m × 30 m in forest land, savannah grassland and cropland and fallow or agricultural land, 100 m × 100 m within settlements and 10 m × 20 m in agroforestry and plantation. A total number of 250 plots (Fig. 4 and Table 4) were surveyed-in total they covered 27.26 ha.

#### Importance Value Index (IVI) analysis

The IVI of a species is the sum of the relative frequency, relative density and relative dominance of the species [52]. Chabi et al. [45] estimated the IVI of the main species when developing biomass allometric models in the same watershed in the North-West of Benin. 84 species were identified during plots surveys. Three variables (DBH, total height of stand tree and wood density (Chabi et al. [45]) of stem wood) were measured from each individual plant of DBH higher or equal than 5 cm. The identified main tree species were *Acacia seyal, Combretum glutinosum, Pterocarpus erinaceus, Anogeisus leiocarpus, Mitragyna inermis, Lannea microcrapa, Ficus sp, Crosopteryx febrifuga, Entada africana, Parkia biglobosa, Vitelaria paradoxa* and *Azadirachta indica* [45].

#### Chemical analysis for the estimation of carbon and nitrogen content of stem wood samples

The main tree species of the different land use/land cover classes were identified based on tree inventory data derived during the first field trip. During the second field trip, stem wood samples from the main tree species were taken and analysed later on with respect to their carbon and nitrogen content. Additionally, diameter at breast height (DBH), tree height and wood density were assessed and used as input for an allometric model fitted to the local conditions [45].

During this second field trip, 277 stem wood samples from 18 tree species were obtained. After wood density estimation samples were re-dried, grinded and weighted. Chemical analysis was done at the Institute of Crop Science and Resource Conservation, within the laboratory of the Department of Plant Nutrition in Germany (Bonn) using the EA3000 model CHNS-O Elemental Analyser (http://www.eurovector.it/).

#### Assessment of aboveground carbon and nitrogen stocks

The methodological approach to calculate the carbon and nitrogen stocks was similar across all LULC of Table 4.

For this purpose as only stand trees species were concerned in this study, the estimation of aboveground carbon and nitrogen stocks was based on the biomass estimation at the tree level using the published equations from Chabi et al. [45] corresponding to each LULC for all tree species, except for two tree species. For the Senegal date palm (*Phoenix reclinata*) and the Asian Palmyra palm (*Borassus flabellifer*) biomass was estimated using the equation from Schroth [53] developed for coconut tree (*Cocos nucifera*) which is a member of the family Arecaceae (palms) such as *Borassus flabellifer* and *Phoenix reclinata*. For the estimation of aboveground biomass of tree species of crop land and fallow and the savannah grassland we also apply the published equations from Chabi et al. [45] corresponding to these two LULC classes. These published equations can be found in the additional file 2 of Chabi et al. [45].

By combining the carbon content of the different tree species or the nitrogen content of the different tree species (Table 1) with the biomass estimated from the allometric models Chabi et al. [45], carbon and nitrogen stocks were estimated at the tree and the plot level (Eqs. 1a; 2a, 3 and 4). When the tree species did not belong to the main tree species of Table 1, we applied the overall mean of carbon and nitrogen content across all species to estimate their carbon and nitrogen stocks (Eqs. 1b, 2b).

1a $$C_{t} = C_{ts} *B_{t}$$

1b $$C_{t} = C_{mc} *B_{t}$$

2a $$N_{t} = N_{ts} *B_{t}$$

2b $$N_{t} = N_{mn} *B_{t}$$

3 $$C_{p} = \mathop \sum \limits_{i = 1}^{n} C_{ti}$$

4 $$N_{p} = \mathop \sum \limits_{i = 1}^{n} N_{ti}$$

where: B_t_, Biomass at the tree level and this is the function of the published equation from Chabi et al. [45]; C_t_, The carbon stock in the dry matter at the tree level; C_ts_, The fraction of Carbon content of the tree species or the percentage of C in the dry matter of the tree species; C_mc_, The mean fraction of carbon content for all 277 wood samples in the case study. C_mc_ equal to 0.4701. The IPCC [42] default value is equal to 0.47. C_mc_ is used when the tree species did not belong to the tree species of the Table 1; N_t_, The nitrogen stock in the dry matter at the tree level; N_ts_, The fraction of Nitrogen content of the tree species or the percentage of N in the dry matter of the tree species; N_mn_, The mean fraction of nitrogen content for all 277 wood samples in the case study. N_mn_ equal to 0.229; Cp, The carbon stock at the plot level; Np, The nitrogen stock at the plot level; n, The total number of tree species in the plot, the index variable i goes from 1 to n.

By combining information from carbon and nitrogen stocks at plot level with the land use/land cover classification (Table 4), carbon as well as nitrogen stocks for each LULC were calculated as mean carbon and nitrogen density (Eqs. 5 and 6), (Tables 2 and 3) times the area of the LULC class (Table 4 and Fig. 5).

5 $$C_{dLULC} = \frac{{\mathop \sum \nolimits_{i = 1}^{np} C_{pi} }}{\text{np}} \pm \varepsilon$$

6 $$N_{dLULC} = \frac{{\mathop \sum \nolimits_{i = 1}^{np} N_{pi} }}{\text{np}} \pm \varepsilon$$

where: C_dLULC_, Carbon density for each LULC expressed in Mg/ha with associated standard error (Ɛ); N_dLULC_, Nitrogen density for each LULC expressed in Mg of N per ha with associated standard error (Ɛ); np, The total number of the plots in each LULC, the index variable i goes from 1 to np; C_pi_, The carbon stock of the plot i; N_pi_, The nitrogen stock of the plot i.

The carbon and nitrogen stocks maps were compiled in ArcGIS 10.2.1 (http://www.esri.com/) and visualized (Figs. 2 and 3).

## Data Availability

We declare available data and material used in the setting of this study.

## Abbreviations

C: carbon
DBH: diameter at breast height
IVI: Importance Value Index
IPCC: Intergovernmental Panel on Climate Change
LUCa: land use category
LULC: land use/land cover
Mg: megagramme
N: nitrogen
REDD+: reducing emissions from deforestation and forest degradation, biodiversity conservation, sustainable forest management and enhancement of forest carbon stocks
SE: standard error
SPI: standardized precipitation index
